# Report on the 4'th scientific meeting of the “Verein zur Förderung des Wissenschaftlichen Nachwuchses in der Neurologie” (NEUROWIND e.V.) held in Motzen, Germany, Nov. 2’nd – Nov. 4’th, 2012

**DOI:** 10.1186/2040-7378-4-22

**Published:** 2012-11-22

**Authors:** Ralf A Linker, Sven G Meuth, Tim Magnus, Thomas Korn, Christoph Kleinschnitz

**Affiliations:** 1 Department of Neurology, Friedrich-Alexander-Universität Erlangen, Schwabachanlage 6, 91054, Erlangen, Germany; 2 Department of Neurology - Inflammatory Disorders of the Nervous System and Neurooncology, Westfälische Wilhelms-University Münster, Domagkstr. 13, 48149, Münster, Germany; 3 Department of Neurology, University Clinic Hamburg-Eppendorf, Martinistr. 52, 20246, Hamburg, Germany; 4 Department of Neurology, Klinikum rechts der Isar, Technische Universität München, Ismaninger Str. 22, 81675, München, Germany; 5 Department of Neurology, University Clinic of Würzburg, Josef-Schneider-Str. 11, 97080, Würzburg, Germany

## Abstract

From November 2^nd^ - 4^th^ 2012, the 4^th^ NEUROWIND e.V. meeting was held in Motzen, Brandenburg, Germany. Again more than 60 participants, predominantly at the doctoral student or postdoc level, gathered to share their latest findings in the fields of neurovascular research, neurodegeneration and neuroinflammation. Like in the previous years, the symposium provided an excellent platform for scientific exchange and the presentation of innovative projects in the stimulating surroundings of the Brandenburg outback. This year’s keynote lecture on the pathophysiological relevance of neuronal networks was given by Christian Gerloff, Head of the Department of Neurology at the University Clinic of Hamburg-Eppendorf. Another highlight of the meeting was the awarding of the NEUROWIND e.V. prize for young scientists working in the field of experimental neurology. The award is donated by the Merck Serono GmbH, Darmstadt, Germany and is endowed with 20.000 Euro. This year the jury decided unanimously to adjudge the award to Michael Gliem from the Department of Neurology at the University Clinic of Düsseldorf (group of Sebastian Jander), Germany, for his outstanding work on different macrophage subsets in the pathogenesis of ischemic stroke published in the *Annals of Neurology* in 2012.

## Summary of the scientific contributions to the NEUROWIND e.V. meeting 2012

### Contributions on stroke and vascular pathology

Mathias Gelderblom from the group of Tim Magnus, Hamburg, reported on the role of the IL-17 axis in a murine model of ischemic stroke. He nicely showed that gamma delta T cell derived IL-17, TNF-α, and CXCL1 are important mediators of neutrophil invasion and tissue damage after an ischemic insult.

Next, Arthur Liesz from the group of Roland Veltkamp, Heidelberg, gave a talk on clonal expansion of T cells after experimental stroke. Via CDR3 spectratyping, he showed monoclonal and oligoclonal TCR expansion patterns especially in the brain at later time points after ischemia.

Katarzyna Winek from the group of Andreas Meisel, Berlin, presented preliminary findings on the link between gut microbiota and cerebral ischemia and vice versa. By using deep sequencing of the gut microbiome, different surgery conditions for ischemia induction and gut decontamination, it might become possible to unveil an influence of the microbiome on brain infarction in the future.

Sweena Chaudhari from Alma Zernecke`s laboratory in Munich focused on the role of the transcription factor HIF-1α in dendritic cells (DC). An increased atherosclerotic plaque growth was observed in a DC specific conditional HIF-1α knockout mouse on a high fat diet. This phenotype is associated with increased IL-12 expression in DC and higher Th1 activation, which is controlled via STAT3.

Golo Kronenberg (group of Matthias Endres, Berlin) employed transient middle cerebral artery occlusion (tMCAO) in combination with the Porsolt forced swim test and citalopram treatment to analyze post-stroke depression in mice. This model allows for probing monoaminergic transmitter mediated effects after cerebral ischemia, both on a behavioral and neurochemical level.

Christoph Harms from Berlin focused on the role of small ubiquitin-like modifiers (SUMO) in neuroprotection during stroke. He reported that in an oxygen glucose deprivation cell culture model, the formation of SUMO2/3 conjugates is part of the cellular stressresponse. This process may be modulated by RNA interference with SUMO2/3 itself or sentrin specific isoproteases (SENP) as deSUMOylating enzymes.

Daniel-Christoph Wagner from Johannes Boltze`s lab in Leipzig induced photothrombotic brain infarctions in a humanized mouse model on the basis of the NOD-SCID-gamma (NSG) mouse. This model may allow for studying the role of the immune system after stroke in a setting closer to the human situation.

In a translational talk, Jens Minnerup from Münster weighed the pros and cons of mononuclear bone-marrow derived cells as a potential new therapeutic option for the treatment of ischemic stroke. However, injection of these cells three hours after the onset of ischemia in a rat tMCAO model had no effect on the clinical outcome or immune parameters.

Peter Kraft from Christoph Kleinschnitz`s group in Würzburg investigated the role of Foxp3 positive regulatory T cells (Treg) in experimental stroke using DEREG mice. Surprisingly, a detrimental rather than a beneficial function of Treg in ischemic stroke was reported which independent of the immune regulatory function of Treg. Instead, Treg-endothelium and Treg-platelet interaction may play a key role in this process thus identifying ischemic stroke as a “thrombo-inflammatory“ disease.

Alexander Venus from the group of Marc Fatar, Mannheim, serially investigated APP 23 („Swedish mutation“) transgenic mice by *in vivo* high-field (9.4T) magnetic resonance imaging (MRI) to visualize amyloid plaques, microbleedings and vasculopathy. These investigations may help to establish the APP 23 model as a suitable tool for further studies on the pathogenesis of cerebral amyloid angiopathy.

In a related approach, Stefanie Schreiber from Klaus Reymann`s group in Magdeburg examined the process of small vessel disease in spontaneously hypertensive stroke prone rats (SHSPR). This model is characterized by lipohyalinosis and microthrombosis including capillary changes as well as a hypercoagulation state already at an early phase of the disease. Moreover, brain changes are obviously paralleled by hypertensive nephropathy.

### Contributions on neuroimmunology

Stefanie Kürten, Cologne, gave an interesting overview on the role of B cells in a mouse model of multiple sclerosis (MS) induced by the immunization with the MP4 fusion protein. A pathological hallmark of this model is the formation of B cell aggregates in the CNS, similar to MS patients. These tertiary lymphatic structures may play a detrimental role in the pathogenesis of the disease.

Stefan Bittner from Sven Meuth`s laboratory in Münster focused on TREK channels in neuroinflammation. The group found that upon experimental autoimmune encephalomyelitis (EAE) TREK1 channel expression is regulated at the blood brain barrier and in neurons thus suggesting a dual role of this molecule in immune cell transmigration and direct neuroprotection.

Nils Schweingruber from the group of Holger Reichardt and Fred Lühder, Göttingen, reported on mechanisms of glucocorticoid action in murine EAE. Using different glucocorticoid receptor knockout mice, he showed that altered migration of (naive and memory) T cells rather than induction of apoptosis is critical for the beneficial effects of the prototypic glucocorticoid compound dexamethasone in a process mediated via CXCR4.

Tobias Goldmann from Freiburg (lab of Marco Prinz) gave a presentation on the role of ubiquitin specific protease 18 (USP 18) in type 1 interferon (IFN) signaling. USP 18 deficient mice suffer from hydrocephalus and microgliosis with increased expression of proinflammatory cytokines and signs of myelin phagocytosis. Further studies were performed to delineate a direct functional interaction of USP 18 with type I IFN receptors.

Christiane Opitz who works in the group of Michael Platten in Heidelberg investigated the tryptophane metabolism in malignant gliomas. The group provided compelling evidence that tryptophane dioxygenase (TDO) is the main enzyme of tryptophane metabolism in glioma cells leading to production of kynurenine. Thus, TDO exerts autocrine effects (on tumor cells) and paracrine effects (on immune cells) via kynurenine-mediated activation of the aryl hydrocarbon receptor.

IIn a presentation on autoimmune-mediated synaptic transmission diseases of the CNS, Christian Geis from Jena reported on stiff person syndrome with anti-amphiphysin antibodies. After intrathecal transfer of these auto-antibodies in rats, there is a GABAergic transmission failure which can be modeled clinically, electrophysiologically and histologically. On a molecular basis, the observed changes may be due to a defect in vesicle endocytosis.

Sina Schröder from the group of Ralf Linker, Erlangen, gave a presentation on the role of non-classical monocytes in MS. In paradigms of mucosal immunity and renal inflammation, classical monocytes are highly efficient phagocytes while non-classical monocytes may exert a patrolling function along blood vessels or in damaged tissue.

Florian Kurschus from Ari Waisman`s group in Mainz presented data on a novel PI3 kinase pathway in EAE which involves IL-22 and which is essential for defining T cell differentiation.

Focusing on vasoactive intestinal peptide (VIP), pituitary adenylate cyclase-activating polypeptide (PACAP), and the cognate VPAC receptors, Dina Hartmann from the group of Björn Tackenberg (Marburg) provided novel data on the neuroendocrine modulation of T cell responses in MS.

IInessa Schwab from Falk Nimmerjahn`s lab in Erlangen talked about the cellular and molecular requirements for the *in vivo* activity of intravenous immunoglobulin (IVIG) therapy. She put special emphasis on Fc-mediated effects and concentrated on the impact of IVIG glycosylation as well as the role of Fcgamma receptor IIb in animal models of rheumatoid arthritis and immune thrombocytopenia (ITP).I In addition, samples from patients suffering from chronic inflammatory demyelinating polyneuropathy (CIDP) treated with IVIG were analyzed.

### Contributions on neurodegeneration

IThomas Koegelsperger explained the impact of transforming growth factor beta 1 (TGFβ1) on synaptic plasticity and neuronal function. In a CNS specific TGFβ1 knockout mouse model there is a mild reduction of neurons and synapses in the hippocampus, astrogliosis and a defect in glutamate reuptake. This phenotype is associated with synaptic and extrasynaptic effects in hippocampal neurons thus highlighting an intricate interplay between the immune system, glial cells and neuronal function.

IMarisa Karow (Munich) highlighted the novel method of direct reprogramming of CNS cells. Her group succeeded in generating (mostly GABAergic) neurons from CD146/Pdgfrβ double positive cells (pericytes) derived from the adult human brain via retroviral transduction with SOX2 and Mash1. If developed further, this promising technology may open novel avenues for the treatment of various neurological diseases.

The work by Johannes Schlachetzki from the laboratory of Jürgen Winkler, Erlangen, addressed the effects of oxidative stress on the posttranslational modification of alpha synuclein (PTM-Syn) in *in vitro* cultures of dopaminergic neurons and microglia.

Finally, Christine Grienberger from Arthur Konnerth`s lab in Munich introduced an *in vivo* study of cortical circuit dysfunction in the APP23/PS45 animal model of Alzheimer`s Disease (AD). The group employed two-photon microscopy in order to image calcium responses in the mouse primary visual cortex. Imaging data were correlated with behavioral tests and analyses of plaque densities as well as beta-amyloid load.

## Competing interests

The authors declare that they have no competing interests.

## Authors’ contributions

RL, SGM, TM, TK, and CK wrote the paper. All authors read and approved the final manuscript.

## Authors’ information

List of speakers at the 4'th scientific meeting of NEUROWIND e.V. (in alphabetical order).

Stefan Bittner, Department of Neurology - Inflammatory Disorders of the Nervous System and Neurooncology, University Clinic of Münster, Germany.

Sweena Chaudhari, Clinic for Vascular and Endovascular Surgery, Technical University Munich, Germany.

Mathias Gelderblom, Department of Neurology, UKE University Clinic of Hamburg, Germany.

Christian Geis, Department of Neurology, University Clinic of Jena, Germany.

Christian Gerloff, Department of Neurology, UKE University Clinic of Hamburg, Germany.

Tobias Goldmann, Institute of Neuropathology, University Clinic of Freiburg, Germany.

Christine Grienberger, Institute of Neuroscience, Technical University Munich, Germany.

Christoph Harms, Center for Stroke Research, Charité, University Medicine of Berlin, Germany.

Dina Hartmann, Department of Neurology, University Clinic of Marburg, Germany.

Marisa Karow, Institute of Physiology, Ludwig-Maximilians University Munich, Germany.

Thomas Koegelsperger, Department of Neurology, Technical University Munich, Germany.

Peter Kraft, Department of Neurology, University Clinic of Würzburg, Germany.

Golo Kronenberg, Department of Neurology, Charité, University Medicine of Berlin, Germany.

Florian Kurschus, Institute for Molecular Medicine, University Medicine of Mainz, Germany.

Stefanie Kürten, Institute for Anatomy I, University of Cologne, Germany.

Arthur Liesz, Department of Neurology, University Clinic of Heidelberg, Germany.

Jens Minnerup, Department of Neurology, University Clinic of Münster, Germany.

Christiane Opitz, Department of Neurooncology, University Clinic of Heidelberg, Germany.

Johannes Schlachetzki, Department of Molecular Neurology, University Clinic of Erlangen, Germany.

Stefanie Schreiber, German Center for Neurodegenerative Disorders (DZNE), Magdeburg, Germany.

Sina Schröder, Department of Neurology, University Clinic of Erlangen, Germany.

Inessa Schwab, Department of Biology, Chair of Genetics, University of Erlangen, Germany.

Nils Schweingruber, Institute for Multiple Sclerosis Research, University of Göttingen, Germany.

Alexander Venus, Department of Neurology, University Clinic of Mannheim, Germany.

Daniel-Christoph Wagner, Fraunhofer Institute for Cell Therapy and Immunology, Leipzig, Germany.

Katarzyna Winek, Department of Neurology, Charité, University Medicine of Berlin, Germany.

